# *Escherichia coli* “TatExpress” strains super‐secrete human growth hormone into the bacterial periplasm by the Tat pathway

**DOI:** 10.1002/bit.26434

**Published:** 2017-10-06

**Authors:** Douglas F. Browning, Kirsty L. Richards, Amber R. Peswani, Jo Roobol, Stephen J.W. Busby, Colin Robinson

**Affiliations:** ^1^ Institute of Microbiology and Infection School of Biosciences University of Birmingham Birmingham UK; ^2^ School of Biosciences University of Kent Canterbury UK

**Keywords:** biopharmaceutical production, human growth hormone, protein secretion, Tat

## Abstract

Numerous high‐value proteins are secreted into the *Escherichia coli* periplasm by the General Secretory (Sec) pathway, but Sec‐based production chassis cannot handle many potential target proteins. The Tat pathway offers a promising alternative because it transports fully folded proteins; however, yields have been too low for commercial use. To facilitate Tat export, we have engineered the TatExpress series of super‐secreting strains by introducing the strong inducible bacterial promoter, *ptac*, upstream of the chromosomal *tatABCD* operon, to drive its expression in *E. coli* strains commonly used by industry (e.g., W3110 and BL21). This modification significantly improves the Tat‐dependent secretion of human growth hormone (hGH) into the bacterial periplasm, to the extent that secreted hGH is the dominant periplasmic protein after only 1 hr induction. TatExpress strains accumulate in excess of 30 mg L^−1^ periplasmic recombinant hGH, even in shake flask cultures. A second target protein, an scFv, is also shown to be exported at much higher rates in TatExpress strains.

## 1 INTRODUCTION

The market for recombinant biopharmaceuticals such as antibody fragments, growth factors, hormones, and other biologically based medicines is estimated to be over $140 billion p.a., with antibody products accounting for a large proportion of these sales (Walsh, 2014). Over a third of currently licensed proteins are produced in *E*. *coli*, where “secretion” out of the cytoplasm to the periplasm is a favored strategy. This minimizes downstream processing (DSP) costs because the target protein can be purified from the relatively simple periplasmic contents, and this strategy avoids debris and DNA contamination, which are serious DSP problems (Balasundaram, Harrison, & Bracewell, 2009). In addition, most biopharmaceuticals contain disulfide bonds, and these can only form in the periplasm in Gram‐negative bacteria.

Efficient *E. coli* secretion‐based systems form the basis for a range of industrial biopharmaceutical production platforms; however, many target proteins fail to be exported to the periplasm because the standard export method (via the Sec pathway) is only capable of transporting proteins in an unfolded state (Natale, Bruser, & Driessen, 2008). Many heterologous proteins pose problems due to rapid folding in the cytoplasm. However, most bacteria possess a second protein export pathway, known as the Tat pathway that has unique capabilities and two major advantages over the standard Sec pathway. First, the Tat pathway exports fully folded proteins, and has already been shown to export a number of “Sec‐incompatible” heterologous molecules (DeLisa, Tullman, & Georgiou, 2003; Thomas, Daniel, Errington, & Robinson, 2001). Moreover, the Tat system can transport substrates up to 150 kDa in size and it even exports some natural substrates in a preassembled dimeric form; Tat therefore has clear potential for export of relatively complex molecules (Rodrigue, Chanal, Beck, Muller, & Wu, 1999). Secondly, the Tat pathway has an inbuilt “quality control” system, whereby it preferentially exports correctly folded proteins—it has been shown to secrete a range of heterologous proteins, including several biopharmaceuticals (Alanen et al., 2015; Matos et al., 2014), yet quantitatively reject virtually every misfolded protein tested to date (DeLisa et al., 2003; Matos, Robinson, & Di Cola, 2008; Richter & Bruser, 2005; Robinson et al., 2011). It thus has potential for the export of correctly folded, highly active target proteins with minimal heterogeneity; that is, it simultaneously provides both a means of exporting the product to the periplasm and increasing product “quality,” thereby decreasing DSP costs.

To date, the Tat system has not been used for industrial secretion‐based strategies, primarily because product yields have been low. This is partly due to the low abundance of the Tat apparatus when compared to the Sec system, which in turn reflects the fact that relatively few proteins are naturally transported by the Tat system (Tullman‐Ercek et al., 2007). It has been shown that over‐expression of the TatABC proteins from a second plasmid can boost export of a heterologous target protein (Matos et al., 2012), but the use of dual plasmids has clear disadvantages for industrial applications (Barrett, Ray, Thomas, Robinson, & Bolhuis, 2003). Here, we show that over‐expression of the *tatABC* genes from the chromosome leads to a major enhancement of Tat export capacity, yielding strains that have clear potential for industrial applications.

## 2 METHODS

### 2.1 Bacterial strains, growth conditions, plasmids, and primers

The bacterial strains and plasmids used in this work are listed in Table 1 and oligonucleotides are listed in Supplementary Table S1. Standard methods for cloning and manipulating DNA fragments were used throughout (Sambrook & Russell, 2001). Derivatives of pDOC‐K (Lee et al., 2009) and pCP20 (Cherepanov & Wackernagel, 1995) were maintained in host cells using media supplemented with 100 μg ml^−1^ ampicillin, pACBSR (Herring, Glasner, & Blattner, 2003) was maintained with 30 μg ml^−1^ chloramphenicol and pEXT22 (Dykxhoorn, St Pierre, & Linn, 1996) derivatives with 50 μg ml^−1^ kanamycin. All bacteria were cultured in LB medium (Sigma, Gillingham, Dorset, UK).

**Table 1:** Strains and plasmids used in this work

Strains/plasmids	Description	Source/reference
W3110	*E. coli* K‐12 strain. F^−^ λ^−^ IN(rrnD‐rrnE)1 *rph‐1*	Hayashi et al. (2006)
TatExpress W3110 Km1	W3110 carrying a kanamycin resistance cassette and the ptac promoter upstream of *tatABCD*	This work
TatExpress W3110	W3110 carrying a ptac promoter upstream of *tatABCD*	This work
BL21	*E. coli* B strain. *fhuA2 [lon] ompT gal [dcm] ΔhsdS*	*New England Biolabs, Hitchin, Hertfordshire, UK*
TatExpress BL21 Km	BL21 carrying a kanamycin resistance cassette and the *ptac* promoter upstream of *tatABCD*	This work
TatExpress BL21	BL21 carrying a *ptac* promoter upstream of *tatABCD*	This work
MC4100	Ara^R^, F2 araD139 DlacU169 rpsL150 relA1 flB5301 deoC1 ptsF25 rbs^R^	Barrett et al. (2003)
ΔtatABCDE	MC4100 strain lacking *tatABCED* genes, Ara^R^	Barrett et al. (2003)
pDOC‐K	Gene doctoring plasmid carrying ampicillin and kanamycin resistance markers	Lee et al. (2009)
pDOC‐ubiB	pDOC‐K carrying the *ubiB* homology region	This work
pDOC‐ubiB/tatA	pDOC‐K carrying the *ubiB* and *tatA* homology regions	This work
pDOC‐TatExpress	pDOC‐K carrying the *ubiB* and *tatA* homology regions with the *ptac* promoter upstream of *tatA*	This work
pACBSR	Gene recombineering plasmid which expresses the λ‐Red and I‐SceI endonuclease genes and carries chloramphenicol resistance	Herring et al. (2003)
pCP20	Temperature sensitive recombineering plasmid which expresses the FLP recombinase and carries ampicillin and chloramphenicol resistance	Cherepanov and Wackernagel (1995)
pEXT22	Protein over‐expression vector carrying kanamycin resistance	Dykxhoorn et al. (1996)
pEXT22/ tatABC	pEXT22 expressing TatABC	Barrett et al. (2003)
pKRK7	pEXT22 expressing TorA‐hGH‐6His	This work

### 2.2 Construction of TatExpress strains

**Figure 1:**
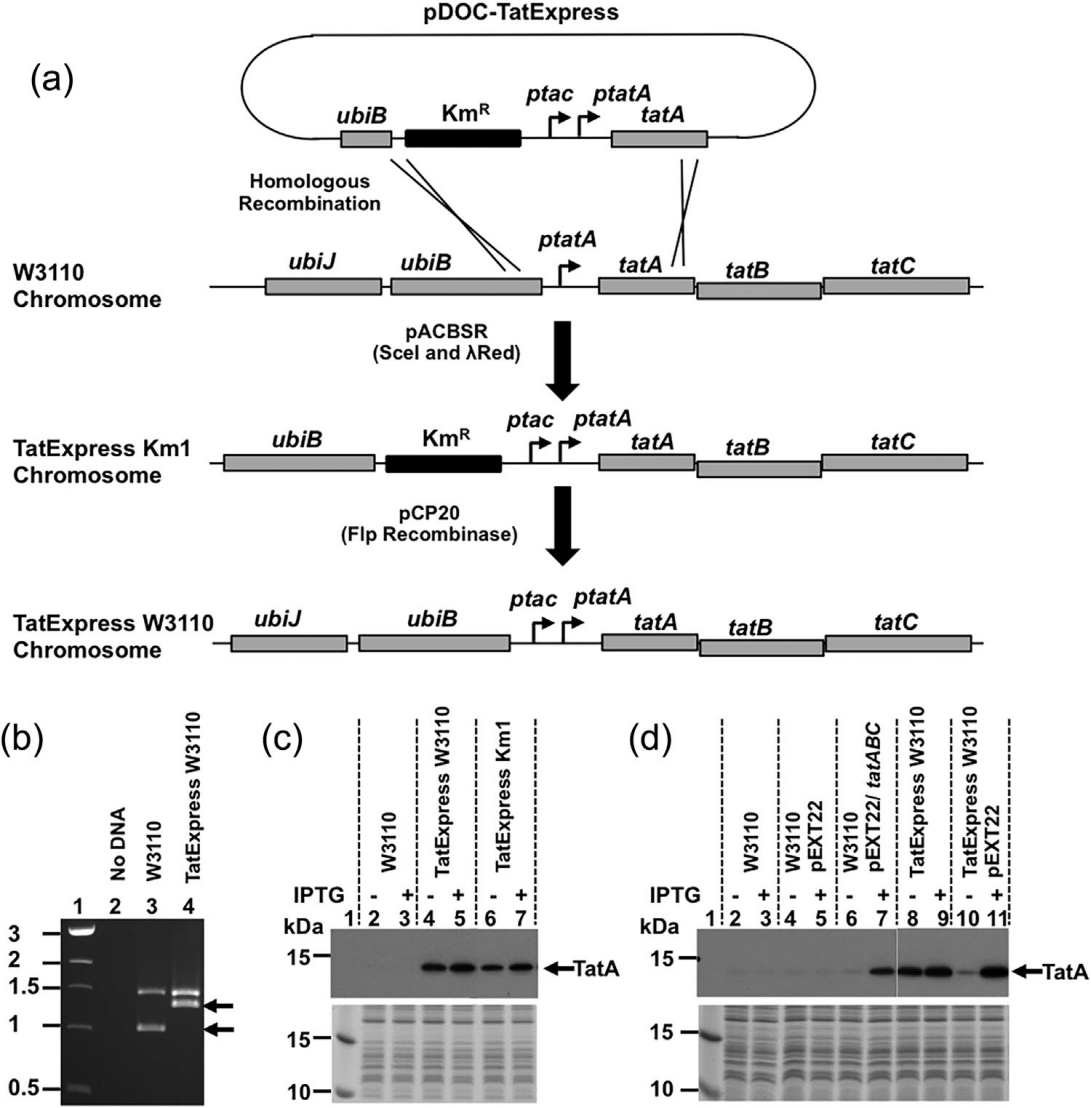
Construction of the TatExpress strains. (a) The panel shows the construction of the *E. coli* K‐12 TatExpress W3110 strain, using gene doctoring (Lee et al., 2009). Plasmid pDOC‐TatExpress was transformed into *E. coli* W3110 pACBSR and after induction of the SceI meganuclease and *λred* gene expression (encoded for by pACSBR), the TatExpress gene cassette was transferred onto the W3110 chromosome by homologous recombination. The resultant TatExpress Km1 strain possesses a kanamycin resistance cassette and the *ptac* promoter upstream of *tatA* and, thus, *tatABCD* operon expression is controlled by *ptac* and the *tatA* promoter (*ptatA*). The kanamycin resistance cassette was removed by transforming cells with pCP20, expressing the Flp recombinase, to generate the TatExpress W3110 strain; (b) PCR analysis of the TatExpress W3110 strain. The panel shows an agarose gel of PCR products in which the *ubiB‐tatA* region was amplified from wild‐type (WT) *E. coli* W3110 and TatExpress W3110; (c) Western blot analysis of W3110 TatExpress strains. The panel shows a Western blot (top panel) and Coomassie blue strained SDS–PAGE gel (bottom panel) of normalized total cell protein from various W3110 strains; (d) Western blot analysis of W3110 and TatExpress W3110, carrying derivatives of plasmid pEXT22. The panel shows a Western blot (top panel) and Coomassie blue strained SDS–PAGE gel (bottom panel) of normalized total cell protein from W3110 and TatExpress W3110 strains, carrying empty pEXT22 vector or pEXT22*/ tatABC*. In both (b) and (c) cells were grown in the presence (+) or absence (−) of 1 mM IPTG and Western blots were probed with anti‐TatA antiserum. Samples were calibrated by loading Page Ruler Plus prestained markers in lane 1 (Thermo Scientific, Waltham, MA) and the location of TatA is indicated by an arrow

To construct the TatExpress series of strains, in which *ptac* is inserted upstream of the *tatA* promoter, gene doctoring methodology was used (Lee et al., 2009) (Figure 1). Initially, the *ubiB* homology region was amplified using PCR with primers UbiBFw and UbiRev and W3110 genomic DNA as template. Purified PCR product was restricted with EcoRI and HindIII and cloned into the pDOC‐K to generate pDOC‐*ubiB*. The *tatA* homology region, which carries the *tatA* promoter and the entire *tatA* open reading frame, was amplified using PCR with primers TatAFw and TatARev and W3110 genomic DNA template. PCR product was digested with NdeI and SpeI and cloned into pDOC‐ubiB, generating pDOC‐*ubiB*/*tatA*. Finally the *ptac* promoter from plasmid pEXT22 (Dykxhoorn et al., 1996) was amplified by PCR, using primers ptacXhoI and ptacNdeI and the product was restricted with XhoI and NdeI and cloned into pDOC‐ubiB/tatA to generate the gene doctoring plasmid pDOC‐TatExpress. This places the *ubiB* homology region and the *ptac*‐*tatA* regions between the kanamycin resistance cassette, encoded by the plasmid (Figure 1). The pDOC‐TatExpress plasmid was transformed into the *E. coli* K‐12 strain W3110, also carrying the gene doctoring plasmid pACBSR, and gene doctoring was carried out as detailed in (Lee et al., 2009). Kanamycin resistant colonies were isolated and the presence of the TatExpress expression cassette was confirmed in the TatExpress Km1 strain, using PCR with primers ubiBFw(check) and tatBrev(check). The kanamycin resistance cassette was removed by transforming candidates with plasmid pCP20 to generate the strain TatExpress W3110. The *ubiB*/*tatA* region of kanamycin sensitive candidates was amplified by PCR, using primers UbiBFw(check) and tatBRev(check), and the purified PCR products were sequenced to confirm strain construction. P1 transduction was used to transfer the TatExpress cassette, still carrying its kanamycin resistance gene marker, from *E. coli* K‐12 TatExpress Km1 to *E. coli* BL21 (Thomason, Costantino, & Court, 2007). Strain validation and the removal of the kanamycin resistance cassette to generate strain TatExpress BL21 was as carried out as detailed above.

### 2.3 Construction of plasmids for protein over‐expression

Derivatives of the low copy number plasmid pEXT22 were used for protein over‐expression, and the pEXT22/hGH construct was created by overlap extension PCR cloning (Bryksin & Matsumura, 2010). Primers pEXT22Up and pEXT22Down (Supplementary Table S1) were used to PCR amplify the target gene of interest from the pET23 based plasmid described in our previous work (Alanen et al., 2015). The purified PCR product was then used as a template in overlap extension PCR to generate the pEXT22/hGH plasmid described in this study (Table 1). The protein amino acid sequence of the expressed TorA‐hGH protein and mature hGH product are detailed in Supplementary Figure S1.

### 2.4 Protein over‐expression

Bacterial cultures were grown at 30°C with shaking in 100 ml of LB medium, supplemented with 50 μg ml^−1^ kanamycin, until an OD_600_ = 0.5 absorbance units (AU), after which protein overexpression was induced by adding either 10 or 100 µm IPTG (isopropyl β‐D‐1‐thiogalactopyranoside). Samples were taken for fractionation prior to induction, and at 1, 2, 3, 4, and/or 5 hr post induction. Periplasmic (P) fractions were collected using an EDTA/lysozyme/cold osmotic shock method previously described (Pierce, Turner, Keshavarz‐Moore, & Dunnill, 1997; Randall & Hardy, 1986) and spheroplasts were further fractionated into cytoplasmic (C) and membrane/insoluble (M) fractions as described in (Pierce et al., 1997).

### 2.5 Sample preparation, protein detection, and quantitation

The preparation of normalized protein samples were carried out as detailed in our previous work (Alanen et al., 2015; Browning et al., 2013). Protein samples were resolved by reducing SDS–PAGE and analyzed using Coomassie blue staining and Western blotting. For Western blotting *E. coli* TatA protein was detected using anti‐TatA antiserum raised in rabbit and an anti‐rabbit‐HRP secondary antibody (Promega, Southampton, Hertfordshire, UK), and detection of C‐terminal‐His_6_ tagged proteins using anti‐His_6_ (C‐terminal) raised in mouse (Invitrogen, Life Technologies Ltd, Paisley, Renfrewshire, UK) and an anti‐mouse‐HRP secondary antibody (Promega). Blots were developed using Clarity Western ECL substrate (Bio‐Rad, Hemel Hempstead, Hertfordshire, UK) and images collected using a Bio‐Rad ChemiDoc XRS+ molecular imager. All gels and blots shown are representative. Quantitation was carried out using Bio‐Rad Image Lab software and calculated using an average of two gels or blots (within linear range) of biological triplicates against a standard curve of purified hGH‐His_6_. Periplasmic hGH was assayed using a hGH ELISA kit as per the manufacturer's protocol (Roche Diagnostics, Charles Ave, Burgess Hill, West Sussex RH15 9RY). Standardized OD10 periplasmic fractions were diluted 1:500,000 using PBS and absorbance was read using a BMG Labtech Spectrostar microplate reader at 405 nm, with a reference wavelength at 490 nm. Concentrations were calculated from two independent experiments and all samples measured in duplicate, and used to calculate average periplasmic yield (in mg/L) by factoring in culture OD readings at 600 nm.

## 3 RESULTS

### 3.1 Construction of TatExpress strains of *E. coli*

Previously, we demonstrated that overexpression of TatABC from the low copy number plasmid pEXT22/*tatABC* yielded a mild improvement in the secretion of a recombinant protein bearing a Tat peptide (Branston, Matos, Freedman, Robinson, & Keshavarz‐Moore, 2012). In order to minimize the use of antibiotics, and the metabolic burden on cells, we engineered a plasmid‐free system. Thus, we introduced the strong *tac* promoter (*ptac*), which is regulated by the LacI repressor and IPTG (isopropyl β‐D‐1‐thiogalactopyranoside), upstream of the chromosomal *tatABCD* operon in *E. coli* strains W3110 and BL21, using gene doctoring methodology (Lee et al., 2009). To accomplish this we generated the plasmid pDOC‐TatExpress, which carries *ptac* and a kanamycin resistance cassette flanked by the *tatA* and *ubiB* chromosomal sequences (Figure 1a). This expression cassette was crossed onto the W3110 chromosome, and removal of the kanamycin cassette generated strain TatExpress W3110, in which *tatABCD* expression is controlled by both the *ptac* and native *tatA* promoters (*ptatA*) (Figure 1a). PCR analysis and DNA sequencing confirmed correct strain construction (Figure 1b and Supplementary Figure S2) and Western blotting of normalized total protein samples with anti‐TatA antiserum demonstrated that the expression of TatA in TatExpress W3110 was considerably higher than wild‐type (WT) W3110 (Figure 1c) and exceeded that produced by W3110 carrying pEXT22/*tatABC* (Figure 1d, lanes 6 and 7). IPTG‐dependent control of TatA expression in TatExpress W3110 was also achieved by providing the *lacI^q^* gene, which is carried by the empty pEXT22 vector (Figure 1d, lanes 10 and 11). Similar high‐level expression of TatA was also achieved with the equivalent BL21 strain TatExpress BL21 (Supplementary Figure S3).

**Figure 2:**
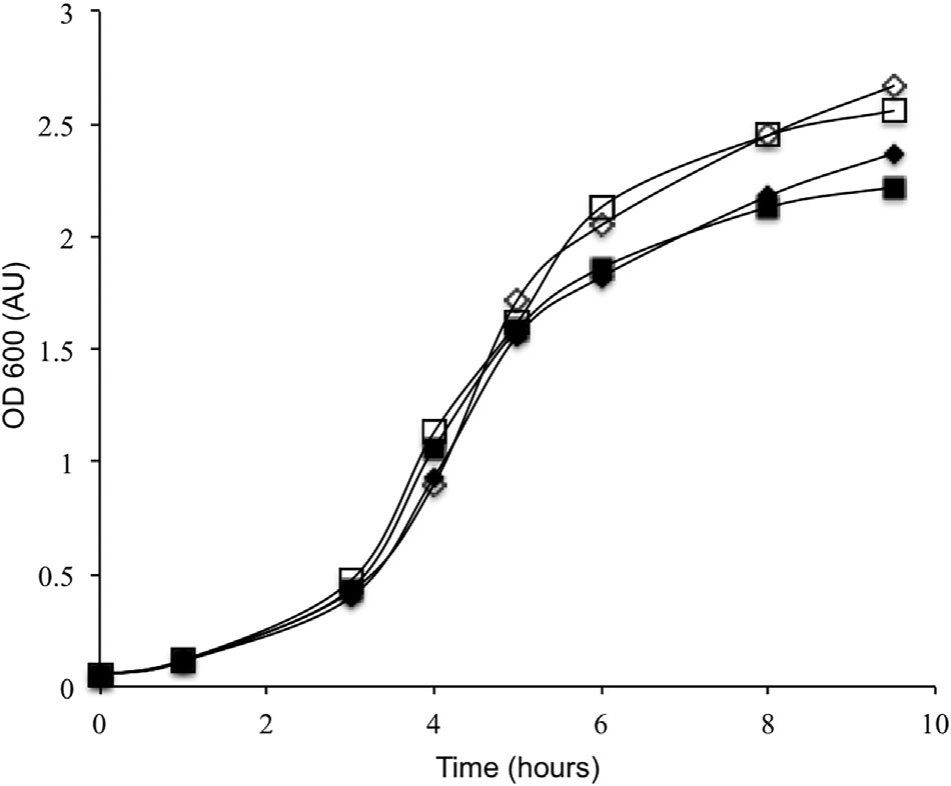
Growth characteristics of W3110 WT and TatExpress strains. The figure shows representative growth curves of W3110 WT and TatExpress strains. All bacterial strains carry the pKRK7 plasmid encoding TorA‐hGH and were grown in 50 ml of LB medium with shaking at 37°C. Induction took place at the 3 hr growth point (approximately 0.5 AU). The graphs are for uninduced W3110 WT and TatExpress (white diamonds and white squares, respectively), and induced W3110 WT and TatExpress (black diamonds and squares, respectively)

Growth of both TatExpress derivatives was indistinguishable from their respective WT strains in shake‐flask cultures using standard LB medium (Figure 2). Similarly, the TatExpress strains showed no growth inhibition in tests using a variety of other media (data not shown). Thus, we have engineered a series of industrially relevant *E. coli* strains to express *tatABCD* to high levels. Additional experiments identified the optimal IPTG concentration for export of hGH in each strain (data not shown), and the strains’ export capacities were tested using a construct in which mature hGH was expressed with an N‐terminal TorA signal peptide and a C‐terminal hexa‐histidine tag (His_6_) from a pEXT22 based plasmid.

### 3.2 TatExpress strains exhibit enhanced export of hGH and an scFv

**Figure 3:**
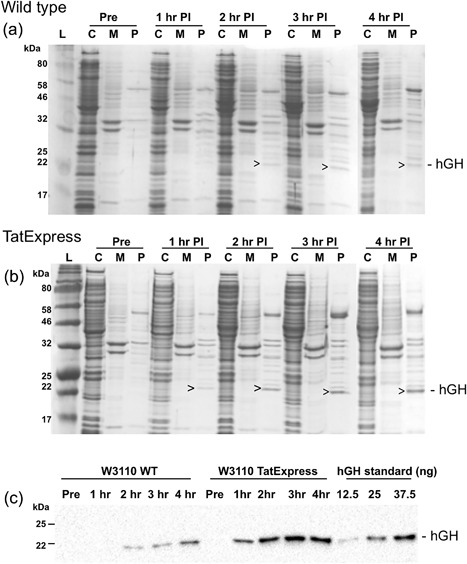
Tat‐dependent export of TorA‐hGH in W3110 and TatExpress W3110 cells. Coomassie blue stained 12.5% SDS‐PAGE gels showing the export of mature hGH (22 kDa, arrowed) into the periplasm of W3110 wild‐type (a) and TatExpress W3110; (b) cells pre‐ and post‐induction using 10 µm IPTG; (c) Western blot of periplasmic samples from (a) and (b) probed using anti‐C‐terminal‐His_6_ antibody. TatExpress W3110 cells show a >5 increase in exported protein at 4 hr post induction relative to export in wild‐type cells. Labels are as follows: C, cytoplasmic fraction; M, membrane and insoluble fraction; P, periplasmic fraction; Pre, pre‐induction; PI, post‐induction. For each lane a normalized amount of protein has been loaded, equivalent to OD_600_ 0.08 AU total cells (Coomassie blue stained gels), and OD_600_ 0.008 AU (immunoblots)

Figure 3 illustrates the expression and export of TorA‐hGH in WT *E. coli* W3110 cells and the TatExpress W3110 strain; expression was induced using 10 µm IPTG and cells were fractionated immediately prior to induction, and at 1, 2, 3, and 4 hr post induction, into cytoplasm, membrane/insoluble, and periplasm samples (C, M, P). Fractionated samples were analyzed by Coomassie stained reducing SDS–PAGE (Figures 3a and 3b) and by immunoblotting periplasmic samples using anti‐His_6_ antibodies (Figure 3c). The immunoblot (Figure 3c) shows that hGH is efficiently exported to the periplasm in WT cells, as observed previously (Alanen et al., 2015). Export in TatExpress W3110 cells is, however, considerably more efficient, with a >5‐fold enhancement found in TatExpress cells. The Coomassie gels provide a measure of the abundance of the exported protein. In WT cells, the periplasmic hGH is visible after 2 hr of induction (arrow) and by the end of 4 hr induction it is clearly visible within the periplasmic fraction (Figure 3a). However, export of TorA‐hGH is much more efficient in TatExpress W3110 cells and it is notable that, by the end of the 4 hr induction, hGH is the second most abundant protein in the periplasm. The identity of the hGH band was confirmed using mass spectrometry (Supplementary Figure S4). Mature hGH is the second most prominent band after the 61.5 kDa OppA protein, a periplasmic subunit of an oligopeptide ABC transporter required for peptide uptake (and strongly induced by growth in LB media). Quantitation of immunoblots using a hGH‐His_6_ protein standard show that average periplasmic export levels are 14.5 mg L^−1^, with the cultures reaching OD_600_ values of 2.5 AU after 4 hr induction.

**Figure 4:**
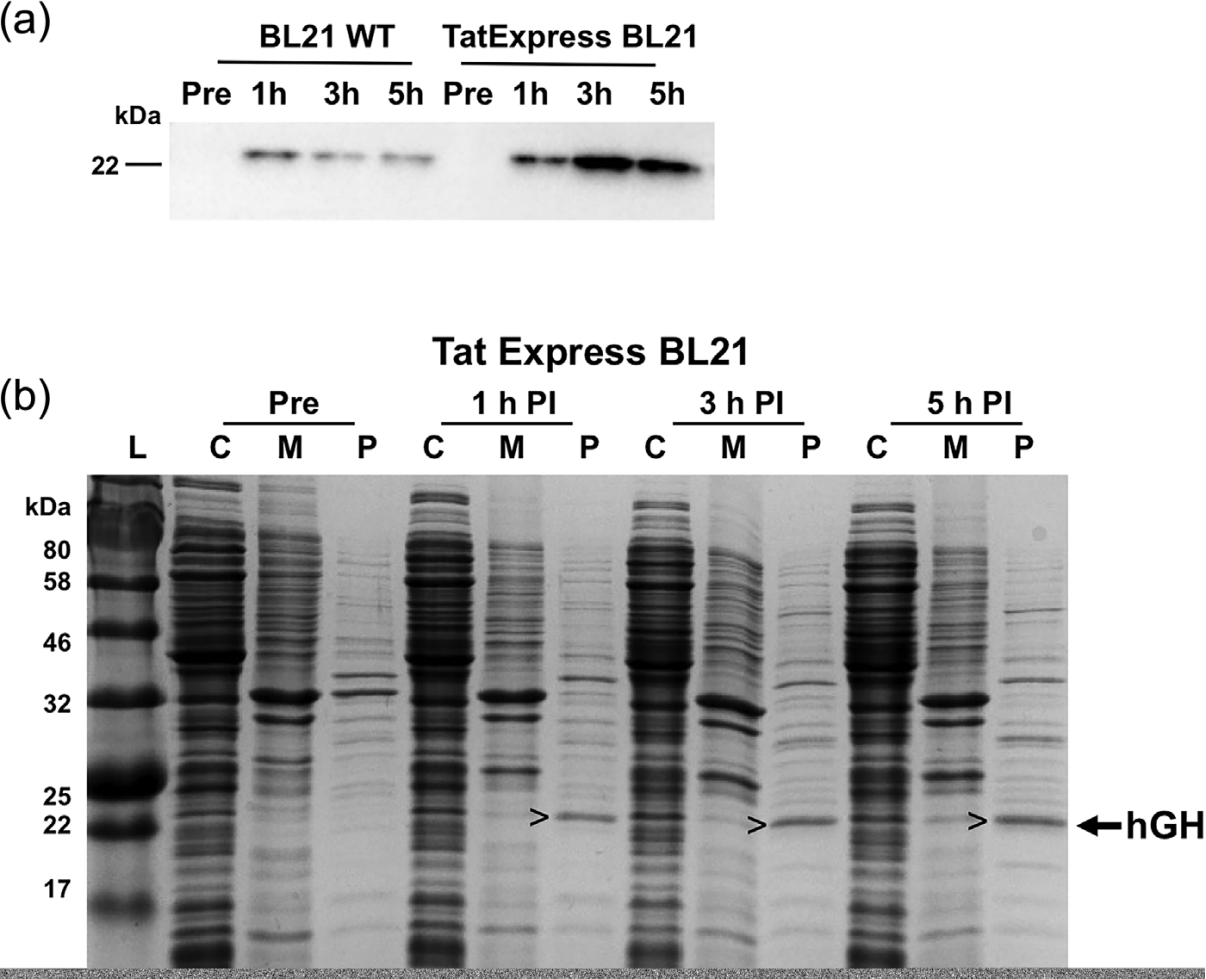
Export of TorA‐hGH in BL21 and TatExpress BL21 cells. (a) immunoblot showing showing the accumulation of mature hGH (22 kDa) in the periplasm of BL21 wild‐type cells and TatExpress BL21 cells upon the induction of protein over‐expression by the addition of 100 µm IPTG. Periplasmic samples were analyzed pre‐induction (Pre) and after 1, 3, and 5 induction; (b) Coomassie‐stained gel of cytoplasmic fractions, membrane and insoluble fractions and periplasmic fractions (C, M, P) from TatExpress BL21 cells expressing TorA‐hGH. Samples were analyzed pre‐induction (Pre) and 1, 3, and 5 hr post‐iduction (PI). For each lane a normalized amount of protein has been loaded, equivalent to OD_600_ 0.08 AU total cells

Similar tests using TatExpress BL21 cells are shown in Figure 4. Here, expression was induced with 100 µm IPTG and cells were analyzed before induction, and at 1, 3, and 5 hr post induction. Immunoblotting analysis of periplasmic fractions (Figure 4a) shows the appearance of mature hGH after 1 hr induction, and the data show that TatExpress cells exhibit a major increase in hGH export (a 4.8‐fold increase was calculated by densitometry). SDS–PAGE analysis of TatExpress BL21 cells (Figure 4b) shows that export is clearly evident at 1 hr post induction, with a substantial periplasmic hGH band visible (arrowed). Export proceeds during the 5 hr induction, and by the end of the induction period hGH is by far the most prominent periplasmic band. Quantitation of the immunoblots shows that the TatExpress BL21 cells export 31** **mg L^−1^ of hGH to the periplasm, with the cultures reaching OD_600_ values of 2.5 AU after 5 hr induction. Separate quantitation using a commercial hGH ELISA test (see section 2) gave a figure of 22 mg L^−1^ hGH.

**Figure 5:**
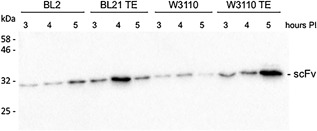
An scFv is exported in significantly higher quantities in TatExpress cells. A construct comprising an scFv bearing a TorA signal peptide was expressed in WT W3110 and BL21 cells, and in the TatExpress versions, as carried out with TorA‐hGH in Figures 3 and 4. After induction for 3 hr, cells were fractionated and blotted as described in Figures 3 and 4

Similar tests were carried out using a second target molecule: a single chain antibody fragment (scFv) that has been previously shown to be efficiently exported by Tat if a TorA signal peptide is attached (Alanen et al., 2015). This construct was expressed in WT W3110 and BL21 strains, and in the TatExpress variants (W3110 TE and BL21 TE) and export assays were carried out after 3, 4, and 5 hr induction. The periplasmic fractions were immunoblotted to detect the export scFv (Figure 5). In some cases the cells exhibit lowered export by the 5 hr time point, which reflects the onset of stress as the cells go into stationary phase, but the data for the 3 and 4 hr time points show a consistent picture: both the W3110 and BL21 TatExpress strains show a very marked enhancement of export when compared with the WT strains.

### 3.3 Optimization of induction protocol is a key factor in the high‐level export capacity of TatExpress strains

**Figure 6:**
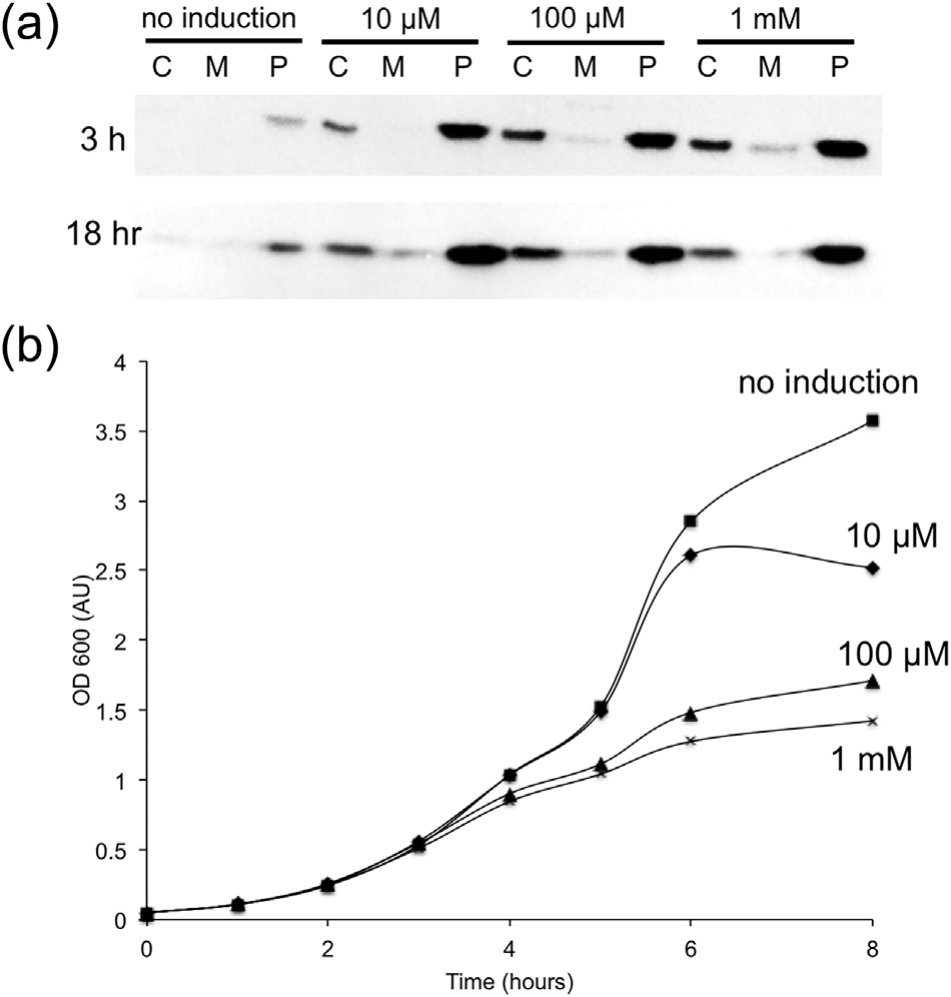
Excessive IPTG concentrations lead to inhibition of growth and reduced export in TatExpress cells. (a) expression of TorA‐hGH in W3110 TatExpress cells was induced by the addition of IPTG at the concentrations shown (after 3 hr growth at approximately 0.5 AU). Cells were fractionated to generate cytoplasm/membrane/periplasm samples and the fractions were immunoblotted as shown in Figure 3; (b) effects of IPTG concentration on growth curves of the cultures; uninduced cells are represented by squares and the growth characteristics of cells induced using 10 µm IPTG (diamonds), 100 µm IPTG (triangles), and 1 mm IPTG (crosses) are illustrated

A significant point to emerge from these studies is that optimal results are only achieved by matching the synthesis of the substrate to the Tat system's export capacity. This is illustrated by Figure 6, which shows the effects of increasing IPTG concentration from 10 µm to 1 mm on export efficiency and growth characteristics of the TatExpress W3110 strain. Figure 6a shows that, while efficient export of hGH to the periplasm is observed at all of the IPTG concentraions, the lowest concentration of IPTG actually yields the highest level of mature hGH in the periplasm. We believe that the higher IPTG concentrations do generate more protein product, as would be expected, but that the “excess” hGH is degraded if the Tat system is unable to export it. hGH appears to be particularly susceptible to degradation by cytoplasmic proteases; Supplementary Figure S5 shows that expression of TorA‐hGH in a different strain, MC4100, leads to efficient export to the periplasm whereas expression in the Δ*tat* strain leads to an almost complete loss of protein. In contrast, cytoplasmic expression of TorA‐scFv is readily apparent in Δ*tat* cells. Figure 6b shows that raising the concentration of IPTG also leads to a progressive inhibition of growth, which is consistent with the excess target protein leading to cell stress.

## 4 DISCUSSION

The Tat system's potential as a biotechnological platform has been reported in several studies but low secretion rates have been a serious barrier to industrial exploitation. To our knowledge, no heterologous protein has been exported by Tat to the extent that it ranks among the major periplasmic proteins by the end of the culture period. In this study we have generated super‐secreting strains by (i) generating strains that stably overexpress the Tat system from the chromosome and (ii) matching target protein expression levels to the Tat secretion capacity of the cell. Our results clearly show that combined high‐level expression of both the Tat system and recombinant hGH protein leads to the formation of inclusion bodies and/or degradation of the precursor protein. Lower level expression from a low copy number plasmid, together with induction using an optimal concentration of IPTG, leads to much more efficient secretion rates. The net result is a platform that exports hGH by the Tat pathway at extremely high rates, to the extent that the target protein is the most abundant periplasmic protein within a few hours of induction. The system is also capable of exporting high levels of other proteins, for example a previously tested single chain antibody fragment (Alanen et al., 2015) at high levels.

This platform has clear uses for large scale protein production in fed‐batch fermentation systems, and future studies will assess its potential in fed‐batch fermentation systems that more closely mimic industrial production processes. However, it should also be noted that even this simple shake‐flask system has the ability to generate quantities of protein (tens of mg) that are sufficient for many purposes.

## AUTHORS’ CONTRIBUTION

C.R., D.F.B., and S.J.W.B. conceived and designed the research program. K.L.R., A.P., J.R., and D.F.B. performed the experiments. C.R., K.LR., and D.F.B. wrote the manuscript with input from all authors.

## Supporting information

Additional Supporting Information may be found online in the supporting information tab for this article.

Supporting Data S1.
