# Supplementary material for: Escherichia coli “TatExpress” strains super‐secrete human growth hormone into the bacterial periplasm by the Tat pathway
Source: Biotechnol Bioeng. 2017 Oct 6;114(12):2828–36. doi: 10.1002/bit.26434 (PMC5698719; doi:10.1002/bit.26434)
Supplement: Supplementary file 1 — Supporting Data S1. [file BIT-114-2828-s001.docx]

***Escherichia coli* ‘TatExpress’ strains super-secrete human growth hormone into the bacterial periplasm by the Tat pathway**

**SUPPLEMENTARY INFORMATION**

Douglas F. Browning^1†^*, Kirsty L. Richards^2†^, Amber R. Peswani^2^, Jo Roobol^2^, Stephen J. W. Busby^1^ and Colin Robinson^2^*

^1^ Institute of Microbiology and Infection, School of Biosciences, University of Birmingham, Birmingham, B15 2TT, UK.

^2^ School of Biosciences, University of Kent, Ingram Building, Canterbury CT2 7NJ, UK

^†^ These authors contributed equally to this work.

* For correspondence:

CR : Tel: +44 (0)1227- 823443. Email: [C.Robinson-504@kent.ac.uk](mailto:C.Robinson-504@kent.ac.uk)

DFB: Tel: +44 (0)121-4145435. Email: [D.F.Browning@bham.ac.uk](mailto:D.F.Browning@bham.ac.uk)

**Running title: Export of biopharmaceuticals by the *E. coli* Tat pathway**

**Supplementary Table I.** DNA Primers used in this work. Primers (all are shown 5' to 3').

| Primer Name | Sequence ^a^ | |
| --- | --- | --- |
| **ptacFw** | GGGGCTCGAGAAGGCGCACTCCCGTTCTGGATAATG | |
| **ptacRev** | GGGGGGGCATATGCTGTTTCCTGTGTGAAATTGTTATCCG | |
| **tatAFwd** | TATCATATGCGGTTGGCGCAAAACACGCTG | |
| **tatARev** | ATAACTAGTTTACACCTGCTCTTTATCGTGGCG | |
| **tatBRev(check)** | CCGCCACAGGCAGTCGTTGCGGCCCCAG | |
| **ubiBFwd** | TATGAATTCTGGACATGTACTGTTAAATCTGTTTAATACG | |
| **ubiBRev** | ATAAAGCTTTCAGCGTGTTTTGCGCCAAC | |
| **ubiBFw(check)** | | CCACCAGATACCAACGTTGAAGAGTTCG |
| **pEXT22Up** | | GAGCTCGGTACCCTACCACAGAGGAACATGTATGAACAATAACGA TCTCTTTC |
| **pEXT22Down** | | CAGAAGCTTGCATGCCTGCAGGTCGACTCTAGAAAATCTAGTCGG ATCTCAGT |

**^a^** Restriction sites are underlined

**Figure legends**

**Supplementary Figure 1.** Nucleotide sequence of the region coding for TorA-hGH-His_6_ (a) and translated polypeptide chain (b). The TorA signal peptide is labelled in green, four a.a. of mature TorA protein in red, hGH in black and the His_6_ tag in blue. An NdeI restriction site is also underlined in (a).

**Supplementary** **Figure 2.** Alignment of the *ubiB-tatABC* region from *E. coli* K-12 strains W3110 and TatExpress W3110. The figure shows the DNA sequence of the *E. coli* K-12 strain W3110 *ubiB-tatABC* region, aligned with the corresponding region from the TatExpress 1 strain. The location of the *tatA* promoter (*ptatA*) and various translation start and stop sites are shown for both sequences. For TatExpress 1 the position of the *ptac* promoter and the restriction sites used to construct pDOC-TatExpress are also indicated.

**Supplementary** **Figure 3.** Western blot analysis of BL21 and TatExpress BL21 strains. The figure shows a Western blot (top panel) and Coomassie blue strained SDS-PAGE gel (bottom panel) of normalised total cell protein from BL21 and TatExpress BL21. Cells were grown in the presence (+) or absence (-) of 1 mM IPTG and Western blots were probed with anti-TatA antiserum. Samples were calibrated by loading Page Ruler Plus prestained markers in lane 1 (Thermo Scientific) and the location of TatA is indicated by an arrow.

**Supplementary Figure 4.** Peptide mass fingerprinting analysis of an excised periplasmic hGH gel band by trypsinisation and MALDI TOF mass spectrometry as previously described in ([Alanen et al., 2015](#_ENREF_1)). Identifiable peptide fragments are underlined in the protein sequence. The peptide matches cover 71.4% of hGH-His_6_, confirming the exported protein band to be hGH.

**Supplementary Figure 5.** TorA-hGH and TorA-scFv were expressed in wild type (WT) MC4100 cells and in a *tat* null mutant MC4100 strain. After induction for 3 h, cells were fractionated into cytoplasm, membrane and periplasm samples and the fractions were immunoblotted using antibodies to the C-terminal His tags. The data show that TorA-hGH is not exported in Δ*tat* cells and the cytoplasmic is barely detectable.

**REFERENCES**

Alanen HI, Walker KL, Lourdes Velez Suberbie M, Matos CF, Bonisch S, Freedman RB, Keshavarz-Moore E, Ruddock LW, Robinson C. 2015. Efficient export of human growth hormone, interferon alpha2b and antibody fragments to the periplasm by the *Escherichia coli* Tat pathway in the absence of prior disulfide bond formation. Biochim Biophys Acta 1853(3):756-63.

**Supplementary** **Figure 1.**

a) ATGAACAATAACGATCTCTTTCAGGCATCACGTCGGCGTTTTCTGGCACAACTCGGCGGCTTAACCGTCGCCGGGATGCTGGGGCCGTCATTGTTAACGCCGCGACGTGCGACTGCGGCGCAAGCGGCGCATATGTTCCCAACCATTCCCTTATCCAGGCTTTTTGACAACGCTATGCTCCGCGCCCATCGTCTGCACCAGCTGGCCTTTGACACCTACCAGGAGTTTGAAGAAGCCTATATCCCAAAGGAACAGAAGTATTCATTCCTGCAGAACCCCCAGACCTCCCTCTGTTTCTCAGAGTCTATTCCGACACCCTCCAACAGGGAGGAAACACAACAGAAATCCAACCTAGAGCTGCTCCGCATCTCCCTGCTGCTCATCCAGTCGTGGCTGGAGCCCGTGCAGTTCCTCAGGAGTGTCTTCGCCAACAGCCTGGTGTACGGCGCCTCTGACAGCAACGTCTATGACCTCCTAAAGGACCTAGAGGAAGGCATCCAAACGCTGATGGGGAGGCTGGAAGATGGCAGCCCCCGGACTGGGCAGATCTTCAAGCAGACCTACAGCAAGTTCGACACAAACTCACACAACGATGACGCACTACTCAAGAACTACGGGCTGCTCTACTGCTTCAGGAAGGACATGGACAAGGTCGAGACATTCCTGCGCATCGTGCAGTGCCGCTCTGTGGAGGGCAGCTGTGGCTTCCATCACCATCATCACCAT

b)

10 20 30 40 50 60

MNNNDLFQAS RRRFLAQLGG LTVAGMLGPS LLTPRRATAA QAAHMFPTIP LSRLFDNAML

70 80 90 100 110 120

RAHRLHQLAF DTYQEFEEAY IPKEQKYSFL QNPQTSLCFS ESIPTPSNRE ETQQKSNLEL

130 140 150 160 170 180

LRISLLLIQS WLEPVQFLRS VFANSLVYGA SDSNVYDLLK DLEEGIQTLM GRLEDGSPRT

190 200 210 220 230 240

GQIFKQTYSK FDTNSHNDDA LLKNYGLLYC FRKDMDKVET FLRIVQCRSV EGSCGFHHHH

HH

TorA signal peptide

4 a.a mature TorA protein

NdeI restriction site

hGH protein

His_6_ tag

**Supplementary Figure 2.**

***ubiB....***

K-12 TGGGTGCCACCAGATACCAACGTTGAAGAGTTCGAATTTGCCATTCGTACGGTCTGTGAA

T1 TGGGTGCCACCAGATACCAACGTTGAAGAGTTCGAATTTGCCATTCGTACGGTCTGTGAA

************************************************************

K-12 CCTATCTTTGAGAAACCGCTGGCCGAAATTTCGTTTGGACATGTACTGTTAAATCTGTTT

T1 CCTATCTTTGAGAAACCGCTGGCCGAAATTTCGTTTGGACATGTACTGTTAAATCTGTTT

************************************************************

K-12 AATACGGCGCGTCGCTTCAATATGGAAGTGCAGCCGCAACTGGTGTTACTCCAGAAAACC

T1 AATACGGCGCGTCGCTTCAATATGGAAGTGCAGCCGCAACTGGTGTTACTCCAGAAAACC

************************************************************

K-12 CTGCTCTACGTCGAAGGGGTAGGACGCCAGCTTTATCCGCAACTCGATTTATGGAAAACG

T1 CTGCTCTACGTCGAAGGGGTAGGACGCCAGCTTTATCCGCAACTCGATTTATGGAAAACG

************************************************************

K-12 GCGAAGCCTTTCCTGGAGTCGTGGATTAAAGATCAGGTCGGTATTCCTGCGCTGGTGAGA

T1 GCGAAGCCTTTCCTGGAGTCGTGGATTAAAGATCAGGTCGGTATTCCTGCGCTGGTGAGA

************************************************************

K-12 GCATTTAAAGAAAAAGCGCCGTTCTGGGTCGAAAAAATGCCAGAACTGCCTGAATTGGTT

T1 GCATTTAAAGAAAAAGCGCCGTTCTGGGTCGAAAAAATGCCAGAACTGCCTGAATTGGTT

************************************************************

K-12 TACGACAGTTTGCGCCAGGGCAAGTATTTACAGCACAGTGTTGATAAGATTGCCCGCGAG

T1 TACGACAGTTTGCGCCAGGGCAAGTATTTACAGCACAGTGTTGATAAGATTGCCCGCGAG

************************************************************

K-12 CTTCAGTCAAATCATGTACGTCAGGGACAATCGCGTTATTTTCTCGGAATTGGCGCTACG

T1 CTTCAGTCAAATCATGTACGTCAGGGACAATCGCGTTATTTTCTCGGAATTGGCGCTACG

************************************************************

K-12 TTAGTATTAAGTGGCACATTCTTGTTGGTCAGCCGACCTGAATGGGGGCTGATGCCCGGC

T1 TTAGTATTAAGTGGCACATTCTTGTTGGTCAGCCGACCTGAATGGGGGCTGATGCCCGGC

************************************************************

***ubiB* stop**

K-12 TGGTTAATGGCAGGTGGTCTGATCGCCTGGTTTGTCGGTTGGCGCAAAACACGC**TGA**TTT

T1 TGGTTAATGGCAGGTGGTCTGATCGCCTGGTTTGTCGGTTGGCGCAAAACACGC**TGA**AAG

*********************************************************

**HindIII**

K-12 ------------------------------------------------------------

T1 CTTGGATCCCGGGTACCTAGGACCGGTCAATTGGCTGGAGCTGCTTCGAAGTTCCTATAC

**XhoI**

K-12 ------------------------------------------------------------

T1 TTTCTAGAGAATAGGAACTTCGGAATAGGAACTAAGGAGGATATTCATATCTCGAGAAGG

K-12 ------------------------------------------------------------

T1 CGCACTCCCGTTCTGGATAATGTTTTTTGCGCCGACATCATAACGGTTCTGGCAAATATT

***ptac* -35 *ptac* -10**

K-12 ------------------------------------------------------------

T1 CTGAAATGAGCTG**TTGACA**ATTAATCATCGGCTCG**TATAAT**GTGTGGAATTGTGAGCGGA

**NdeI**

K-12 ---------------------------------------------------TTTTTTCA**T**

T1 TAACAATTTCACACAAGAAACAG**CATATG**CGGTTGGCGCAAAACACGCTGATTTTTTCA**T**

*********

***ptatA* -35 *ptatA* -10**

K-12 **CGCTC**AAGGCGGGCCGTGTAACG**TATAAT**GCGGCTTTGTTTAATCATCATCTACCACAGA

T1 **CGCTC**AAGGCGGGCCGTGTAACG**TATAAT**GCGGCTTTGTTTAATCATCATCTACCACAGA

************************************************************

***tatA* start**

K-12 GGAACATGT**ATG**GGTGGTATCAGTATTTGGCAGTTATTGATTATTGCCGTCATCGTTGTA

T1 GGAACATGT**ATG**GGTGGTATCAGTATTTGGCAGTTATTGATTATTGCCGTCATCGTTGTA

************************************************************

K-12 CTGCTTTTTGGCACCAAAAAGCTCGGCTCCATCGGTTCCGATCTTGGTGCGTCGATCAAA

T1 CTGCTTTTTGGCACCAAAAAGCTCGGCTCCATCGGTTCCGATCTTGGTGCGTCGATCAAA

************************************************************

K-12 GGCTTTAAAAAAGCAATGAGCGATGATGAACCAAAGCAGGATAAAACCAGTCAGGATGCT

T1 GGCTTTAAAAAAGCAATGAGCGATGATGAACCAAAGCAGGATAAAACCAGTCAGGATGCT

************************************************************

K-12 GATTTTACTGCGAAAACTATCGCCGATAAGCAGGCGGATACGAATCAGGAACAGGCTAAA

T1 GATTTTACTGCGAAAACTATCGCCGATAAGCAGGCGGATACGAATCAGGAACAGGCTAAA

************************************************************

***tatA* stop *tatB* start**

K-12 ACAGAAGACGCGAAGCGCCACGATAAAGAGCAGGTG**TAA**TCC**GTG**TTTGATATCGGTTTT

T1 ACAGAAGACGCGAAGCGCCACGATAAAGAGCAGGTG**TAA**TCC**GTG**TTTGATATCGGTTTT

************************************************************

K-12 AGCGAACTGCTATTGGTGTTCATCATCGGCCTCGTCGTTCTGGGGCCGCAACGACTGCCT

T1 AGCGAACTGCTATTGGTGTTCATCATCGGCCTCGTCGTTCTGGGGCCGCAACGACTGCCT

************************************************************

**Supplementary** **Figure 3.**

**
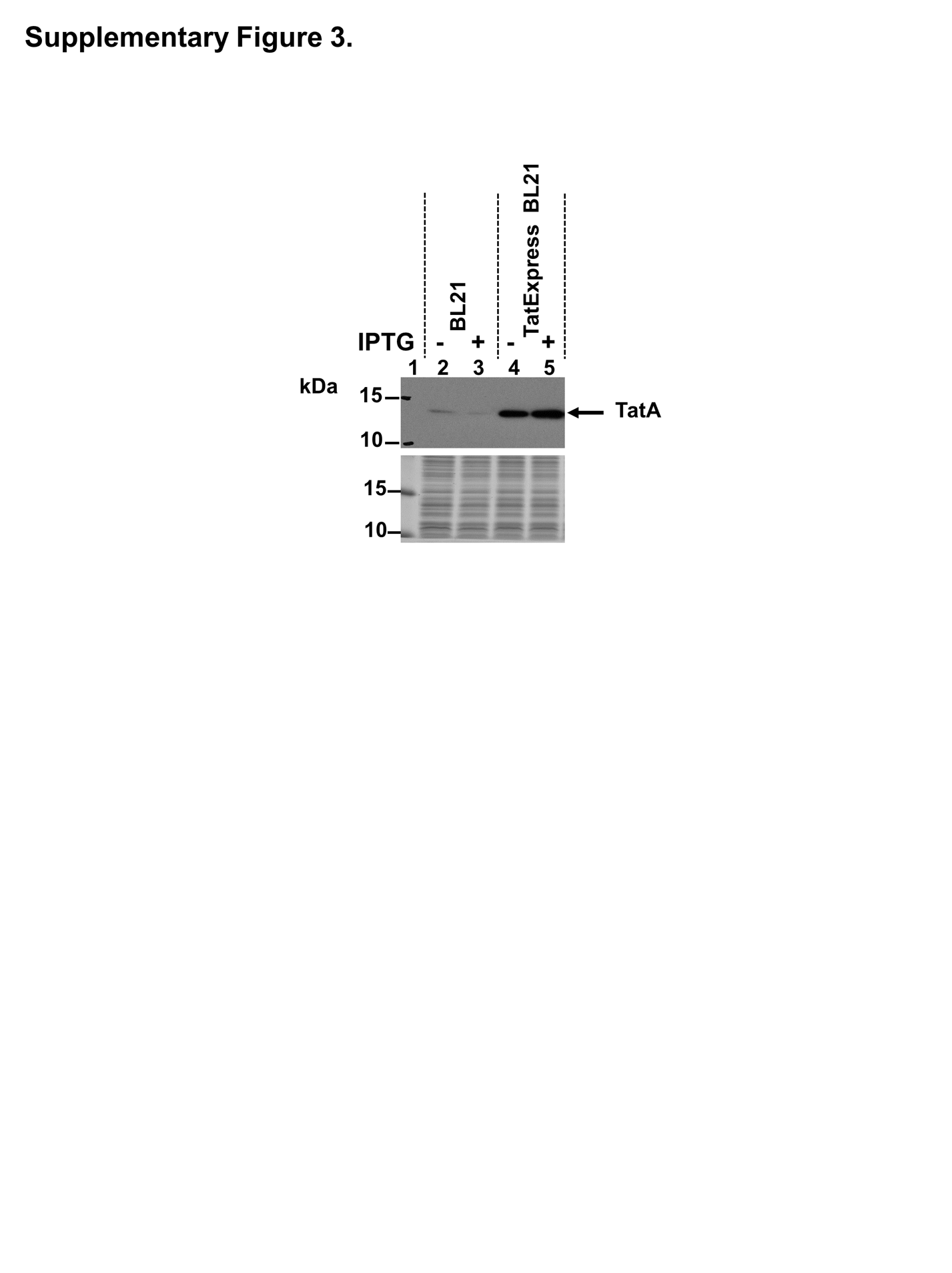
**

**Supplementary** **Figure 4.**

AQAAHMFPTIPLSRLFDNAMLRAHRLHQLAFDTYQEFEEAYIPKEQKYSFLQNPQTSLCS ESIPTPSNREETQQKSNLELLRISLLLIQSWLEPVQFLRSVFANSLVYGASDSNVYDLLKDLEEGIQTLMGRLEDGSPRTGQIFKQTYSKFDTNSHNDDALLKNYGLLYCFRKDMDKVETFLRIVQCRSVEGSCGFHHHHHH

**Supplementary** **Figure 5.**
